# Supplementary material for: Relationship between Middle Cerebral Artery Pulsatility Index and Delayed Neurocognitive Recovery in Patients undergoing Robot-Assisted Laparoscopic Prostatectomy
Source: J Clin Med. 2023 Jan 30;12(3):1070. doi: 10.3390/jcm12031070 (PMC9918143; doi:10.3390/jcm12031070)

**Table S1.** ANOVAs Group (dNCR *vs* No dNCR) × Time (T0 *vs* T1) on each item of SF-36 (n=60). T0= Before surgery; T1= After surgery (2nd day postoperatively). Values are shown as mean (M) ± standard deviation (SD).

| | Patients with dNCR (N=20) | | Patients without dNCR (N=40) | | GroupF (1,58)<br>Time F (1,58)<br>Group × Time F (1,58) | Post-hoc |
|---|---|---|---|---|---|---|
| Item | (T0)<br>M ± SD | (T1)<br>M ± SD | (T0)<br>M ± SD | (T1)<br>M ± SD | | |
| Physical functioning | 78.2 ± 20.7 | 68.2 ± 30.3 | 84.1 ± 17.5 | 70.0 ± 32.1 | Group: 0.39; p= .534<br>Time: 11.17; p= .001<br>Group × Time: 0.32; p=.569 | --- |
| Limitations of role due to physical problems | 60.0 ± 36.3 | 50.0 ± 45.2 | 63.7 ± 35.3 | 48.1 ± 38.1 | Group: 0.01; p=.919<br>Time: 6.87; p=.011<br>Group × Time: 0.33; p= .567 | --- |
| Limitations of role due to emotional problems | 76.6 ± 36.0 | 56.7 ± 40.6 | 75. ± 31.8 | 57.5 ± 38.5 | Group: 0.01; p=.961<br>Time: 13.08; p <.01<br>Group × Time: 0.05; p= .811 | --- |
| Energy-Fatigue | 68.2 ± 15.1 | 56.5 ± 23.0 | 64.2 ± 14.9 | 61.3 ± 11.3 | Group: 0.01; p=.907<br>Time: 12.81; p<.001<br>Group × Time: 4.71; **p=.033** | dNCR T1< dNCR T0; **p= .0008** |
| Emotional well-being | 70.2 ± 14.5 | 59.2 ± 21.1 | 69.3 ± 13.9 | 68.3 ± 12.3 | Group: 1.18; p=.28<br>Time: 14.12; p <.001<br>Group × Time: 9.98; **p=.002** | dNCR T1 < No dNCR T1; **p= .028**<br>dNCR T1 < dNCR T0; **p <.0001** |
| Social functioning | 75.0 ± 20.3 | 63.7 ± 25.9 | 76.2 ± 18.3 | 71.6 ± 18.5 | Group: 0.91; p=.342<br>Time: 7.89; p=.006<br>Group × Time: 1.33; p=.025 | --- |
| Pain | 79.7 ± 23.6 | 49.5 ± 22.3 | 82.5 ± 19.3 | 61.9 ± 20.0 | Group: 2.69; p=.106<br>Time: 58.12; p <.001<br>Group × Time: 2.11; p=.151 | --- |
| General health perception | 54.7 ± 16.2 | 52.5 ± 15.6 | 57.6 ± 15.3 | 56.7 ± 62.6 | Group: 0.88; p=.35<br>Time: 0.82; p=.367<br>Group × Time: 0.12; p=.727 | --- |
| Change in health status | 37.5 ± 17.2 | 42.5 ± 28.2 | 43.1 ± 21.1 | 39.3 ± 20.3 | Group: 0.02 p=.781<br>Time: 0.02; p=.872<br>Group × Time: 1.27; p=.263 | --- |

**Figure S1**. Numerical rate scale (NRS) for pain in patients with dNCR (red line) versus patients without dNCR (blu line) at the following times: at patient's arrival in the recovery room (RR), after 1, 2, 8, 12, 24 and 48 hours. Values are shown as mean ± 95% confidence interval. Interaction F (Group *per* Time) is shown. dNCR, delayed neurocognitive recovery.

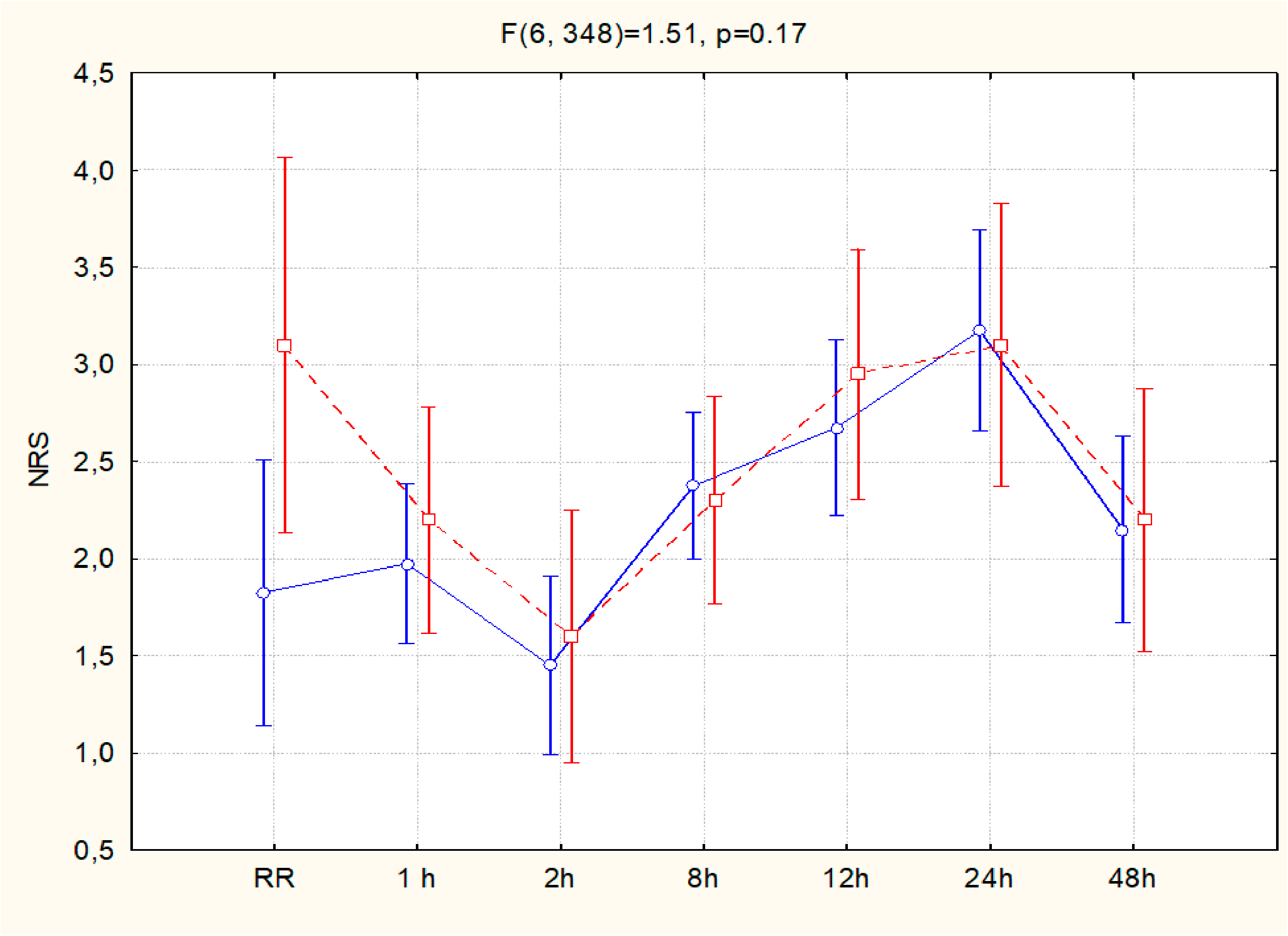

**Figure S2**. Mean arterial pressure (MAP) in patients with dNCR (red line) versus patients without dNCR (blu line) at the following times: before (T1) and after (T2) the induction of anesthesia; 30 minutes (T3) and one hour after the start of ST combined with pneumoperitoneum (T4); before ST and pneumoperitoneum removal (T5); ten minutes after the end of ST and pneumoperitoneum before waking up (T6). Values are shown as mean ± 95% confidence interval. Interaction F (Group *per* Time) is shown. dNCR, delayed neurocognitive recovery. ST, Steep Trendelenburg.

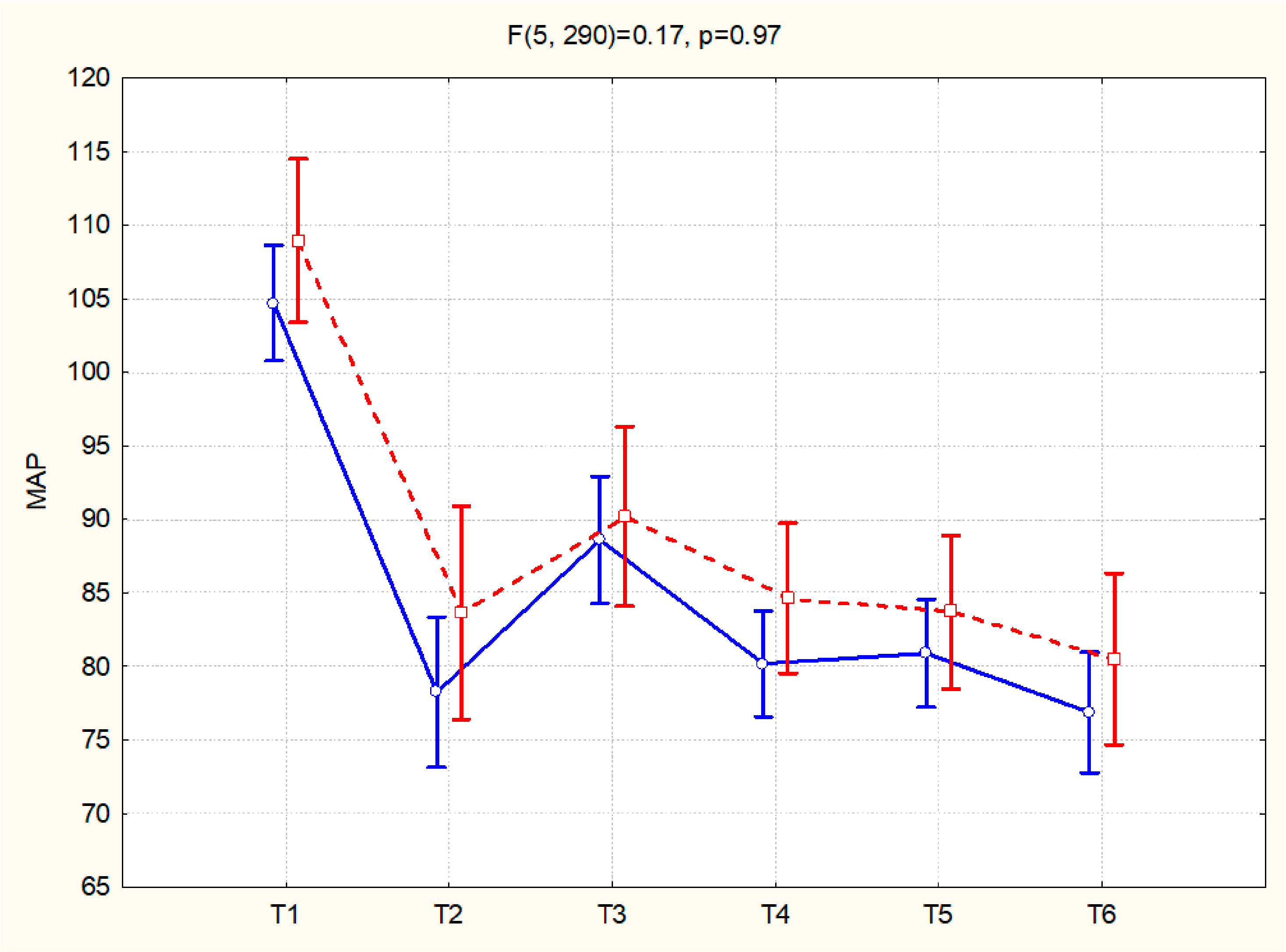

**Figure S3**. Heart rate in patients with dNCR (red line) versus patients without dNCR (blu line) at the following times: before (T1) and after (T2) the induction of anesthesia; 30 minutes (T3) and one hour after the start of ST combined with pneumoperitoneum (T4); before ST and pneumoperitoneum removal (T5); ten minutes after the end of ST and pneumoperitoneum before waking up (T6). Values are shown as mean ± 95% confidence interval. Interaction F (Group *per* Time) is shown. dNCR, delayed neurocognitive recovery. ST, Steep Trendelenburg.

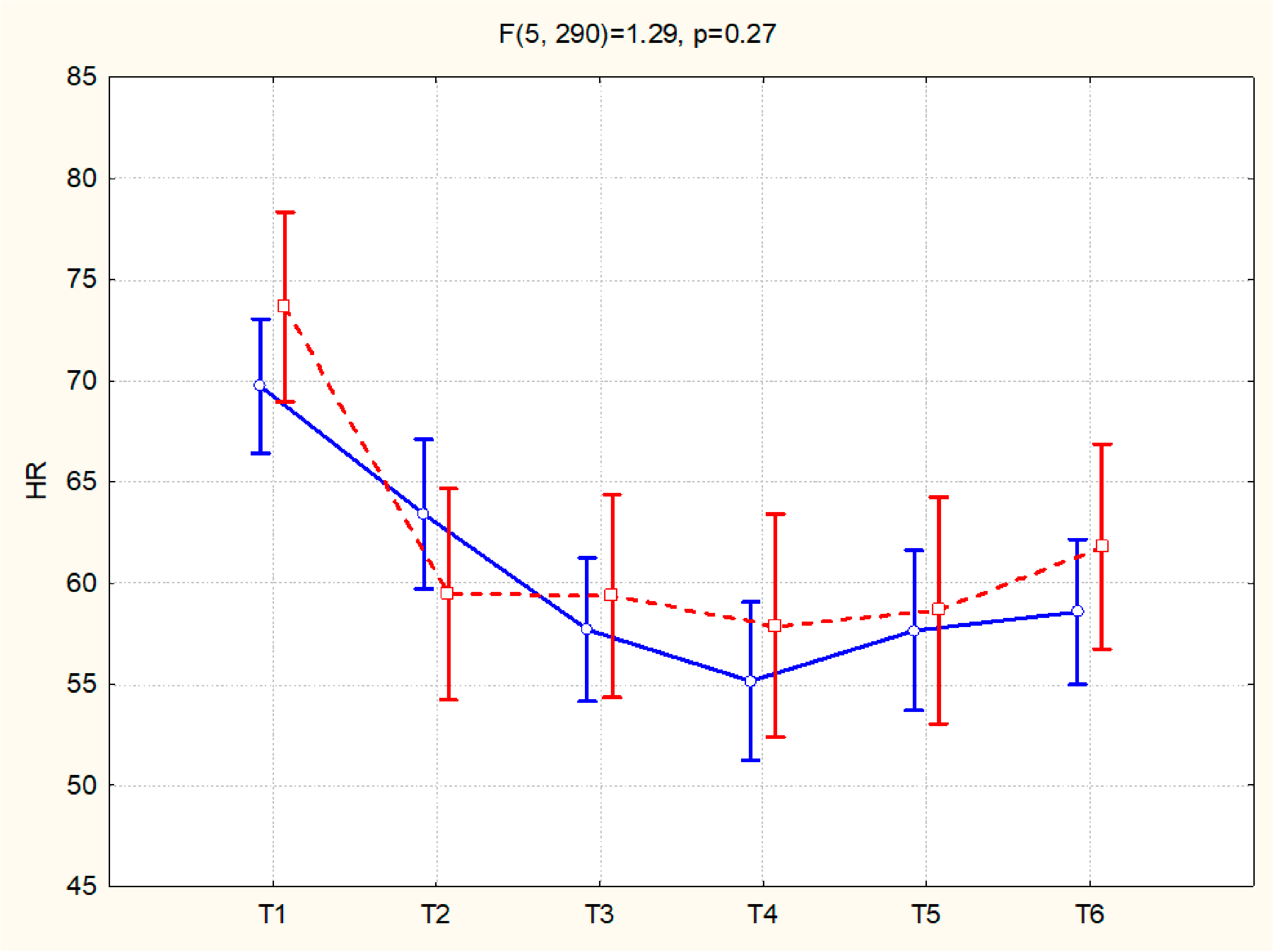

**Figure S4**. End tidal $CO_2$ (mmHg) values in patients with dNCR (red line) versus patients without dNCR (blu line) at the following times: after the induction of anesthesia (T2); 30 minutes (T3) and one hour after the start of ST combined with pneumoperitoneum (T4); before ST and pneumoperitoneum removal (T5); ten minutes after the end of ST and pneumoperitoneum before waking up (T6). Values are shown as mean ± 95% confidence interval. Interaction F (Group *per* Time) is shown. dNCR, delayed neurocognitive recovery. ST, Steep Trendelenburg.

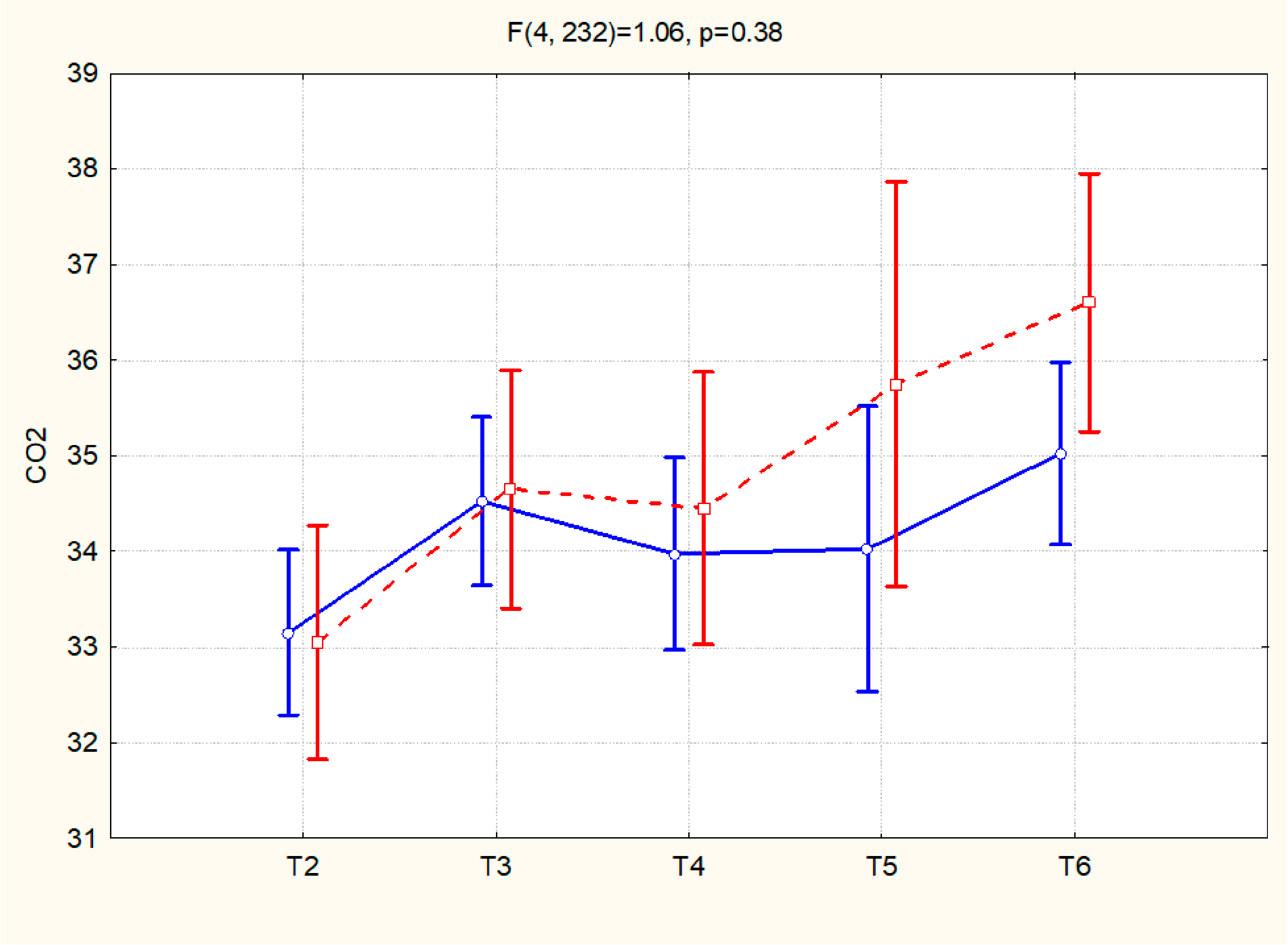

**Figure S5**. Bispectral index (BIS) in patients with dNCR (red line) versus patients without dNCR (blu line) at the following times: before (T1) and after (T2) the induction of anesthesia; 30 minutes (T3) and one hour after the start of ST combined with pneumoperitoneum (T4); before ST and pneumoperitoneum removal (T5); ten minutes after the end of ST and pneumoperitoneum before waking up (T6). Values are shown as mean ± 95% confidence interval. Interaction F (Group *per* Time) is shown. dNCR, delayed neurocognitive recovery. ST, Steep Trendelenburg.

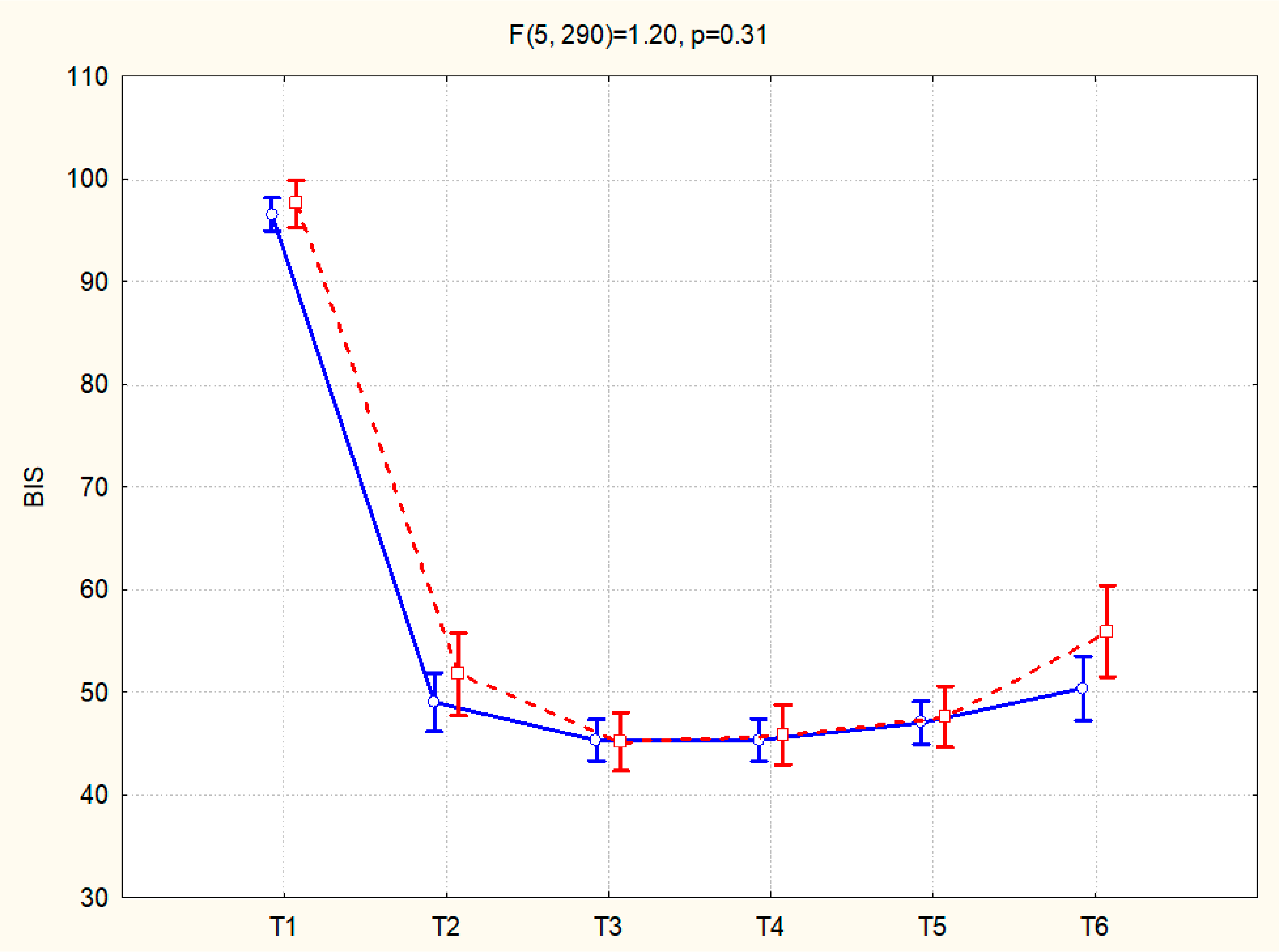

**Figure S6**. Pneumoperitoneum pressure (mmHg) in patients with dNCR (red line) versus patients without dNCR (blu line) at the following times: 30 minutes (T3) and one hour after the start of ST combined with pneumoperitoneum (T4); before ST and pneumoperitoneum removal (T5). Values are shown as mean ± 95% confidence interval. Interaction F (Group *per* Time) is shown. dNCR, delayed neurocognitive recovery. ST, Steep Trendelenburg.

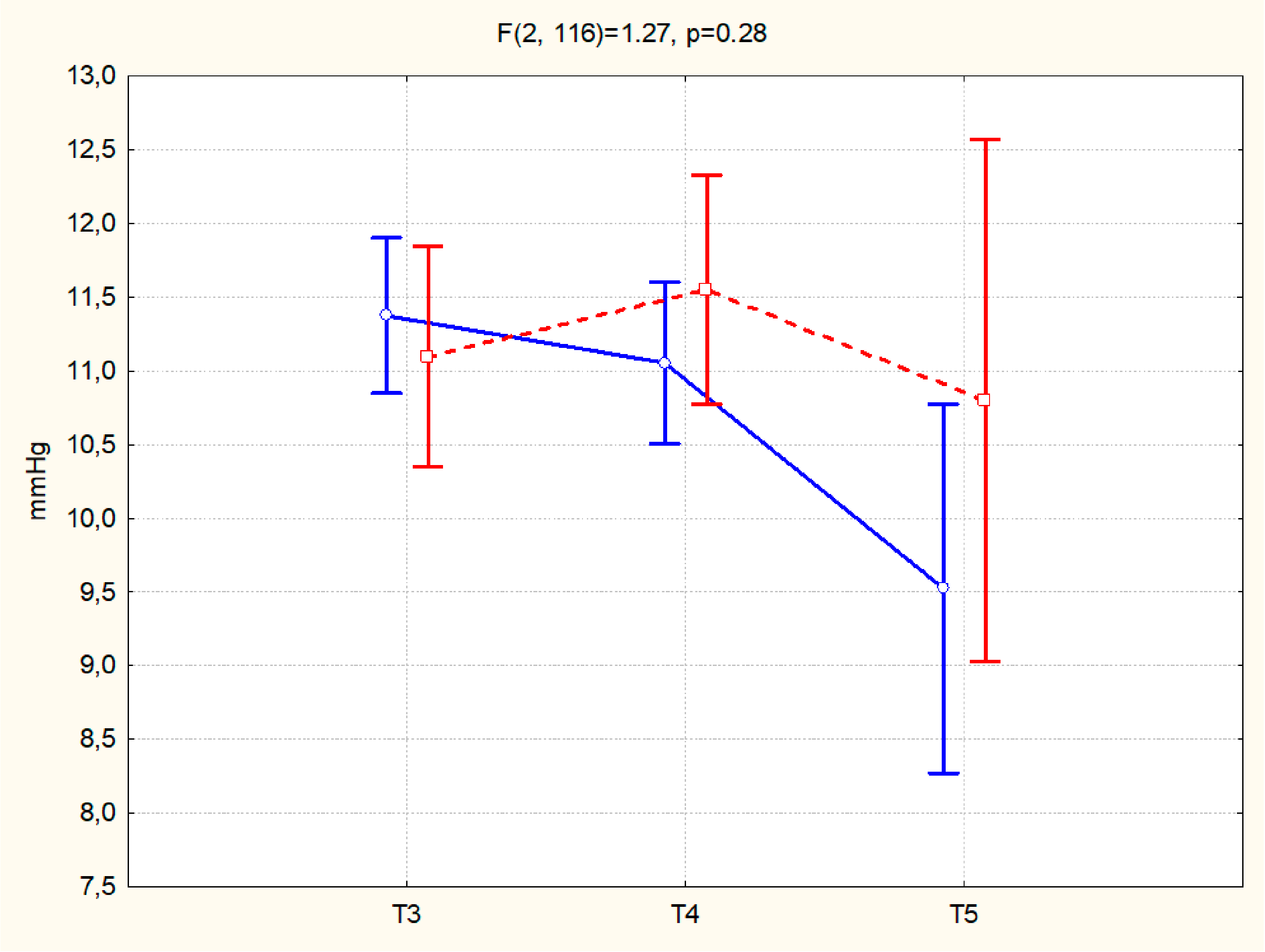

**Figure S7**. Resistivity index in patients with dNCR (red line) versus patients without dNCR (blu line) at the following times: before (T1) and after (T2) the induction of anesthesia; 30 minutes (T3) and one hour after the start of ST combined with pneumoperitoneum (T4); before ST and pneumoperitoneum removal (T5); ten minutes after the end of ST and pneumoperitoneum before waking up (T6). Values are shown as mean ± 95% confidence interval. Interaction F (Group *per* Time) is shown. dNCR, delayed neurocognitive recovery. ST, Steep Trendelenburg.

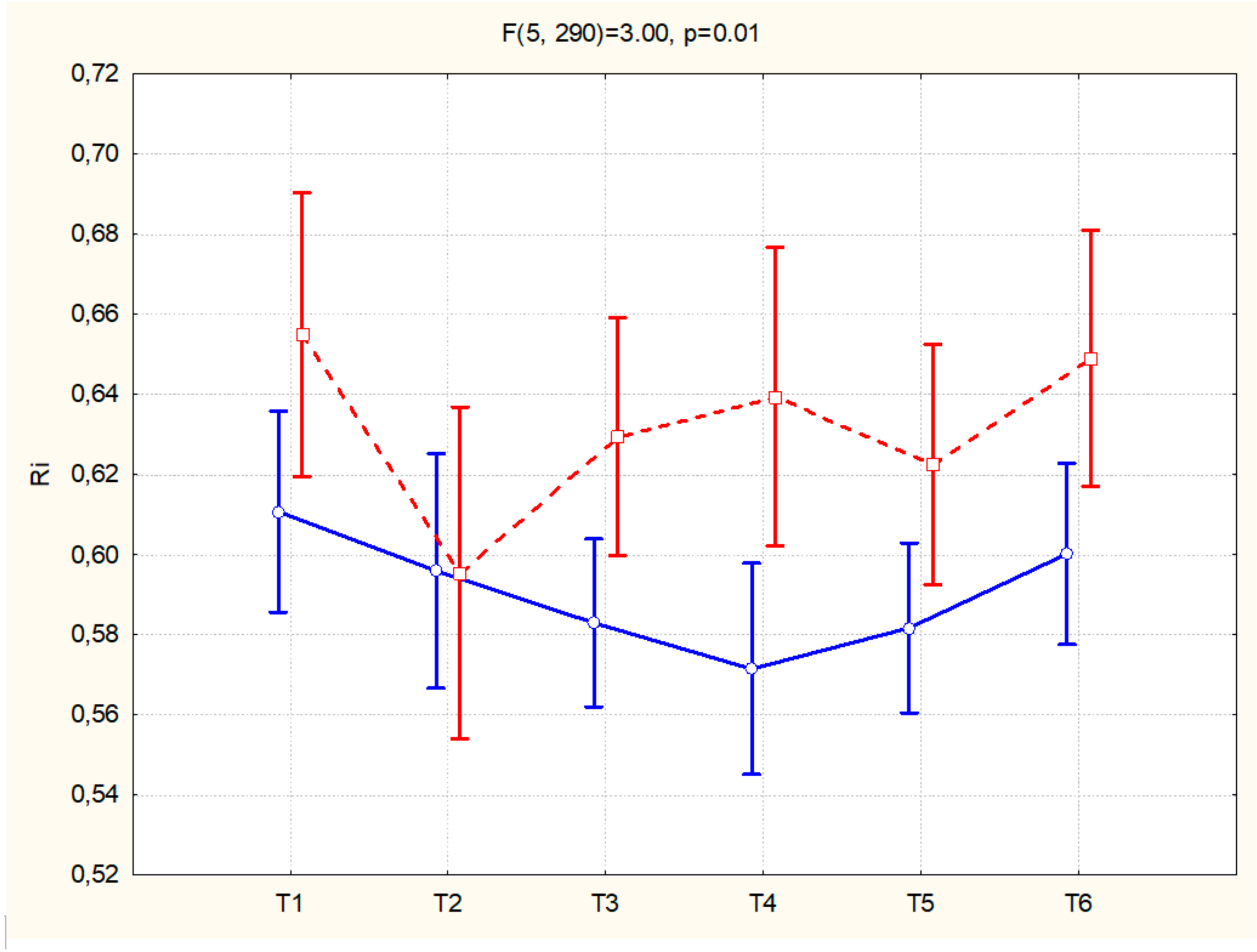

Supplement: Supplementary file 1 [file jcm-12-01070-s001.zip › jcm-2112823-supplementary.pdf]
